# Thyroid Function and Plasma Concentrations of Polyhalogenated Compounds in Inuit Adults

**DOI:** 10.1289/ehp.0900633

**Published:** 2009-05-12

**Authors:** Renée Dallaire, Éric Dewailly, Daria Pereg, Serge Dery, Pierre Ayotte

**Affiliations:** 1 Public Health Research Unit, Centre hospitalier universitaire de Québec-CHUL, Québec, Canada; 2 Nunavik Regional Board of Health and Social Services, Kuujjuaq, Canada

**Keywords:** dioxin-like compounds, hydroxylated metabolites, Inuit, organochlorines, perfluoro-octanesulfonate, polybrominated diphenyl ethers, polychlorinated biphenyls, polyhalogenated compounds, thyroid hormones

## Abstract

**Background:**

Several ubiquitous polyhalogenated compounds (PHCs) have been shown to alter thyroid function in animal and *in vitro* studies. So far, epidemiologic studies have focused on the potential effect of a small number of them, namely, polychlorinated biphenyls (PCBs) and some organochlorines (OCs), without paying attention to other important PHCs.

**Objectives:**

We investigated the relationship between exposure to several PHCs and thyroid hormone homeostasis in Inuit adults from Nunavik.

**Methods:**

We measured thyroid parameters [thyroid-stimulating-hormone (TSH), free thyroxine (fT_4_), total triiodothyronine (tT_3_), and thyroxine-binding globulin (TBG)] and concentrations of 41 contaminants, including PCBs and their metabolites, organochlorine pesticides (OCPs), polybrominated diphenyl ethers (PBDEs), perfluorooctanesulfonate (PFOS), and a measure of dioxin-like compounds, detected in plasma samples from Inuit adults (*n* = 623).

**Results:**

We found negative associations between tT_3_ concentrations and levels of 14 PCBs, 7 hydroxylated PCBs (HO-PCBs), all methylsulfonyl metabolites of PCBs (MeSO_2_-PCBs), and 2 OCPs. Moreover, we found negative associations between fT_4_ levels and hexachlorobenzene Concentrations. TBG concentrations were inversely related to 8 PCBs, 5 HO-PCBs, and 3 OCPs. Exposure to BDE-47 was positively related to tT _3_, whereas PFOS concentrations were negatively associated with TSH, tT_3,_ and TBG and positively with fT_4_ concentrations.

**Conclusion:**

Exposure to several PHCs was associated with modifications of the thyroid parameters in adult Inuit, mainly by reducing tT_3_ and TBG circulating concentrations. The effects of PFOS and BDE-47 on thyroid homeostasis require further investigation because other human populations display similar or higher concentrations of these chemicals.

Thyroid hormones (THs) act on cells of almost all tissues and therefore are involved in several physiologic processes during a life span. In adults, THs are mainly involved in metabolic activities such as protein, lipid, and carbohydrate metabolism and heat generation (Kim 2008). They are also necessary for normal reproductive functions, regulation of heart rate, and gastrointestinal motility, as well as for emotional stability (Bauer et al. 2008; Braverman and Utiger 2005). Disruptions of thyroid function by endogenous (e.g., auto-antibodies) or exogenous (e.g., iodine) factors may produce various subclinical effects (Surks et al. 2004) or direct clinical manifestations. Consequently, there are concerns regarding the ubiquitous background chronic exposure of human populations to polyhalogenated compounds (PHCs), a group of chemicals that include legacy and emerging persistent organic pollutants that have been shown or are suspected to have thyroid-disrupting properties (Brouwer et al. 1995; Chang et al. 2008; Cheek et al. 1999; Hallgren et al. 2001; Kato et al. 2000; van Raaij et al. 1993).

Several epidemiologic studies aimed to evaluate the effects of dioxin-like compounds (DLCs), polychlorinated biphenyls (PCBs), and some organochlorine pesticides (OCPs) on TH homeostasis in adults (Abdelouahab et al. 2008; Hagmar 2003). Effects of these compounds on TSH concentrations were variable, whereas several studies found a negative relationship with total thyroxine (tT_4_) and/or total triiodothyronine (tT_3_), without a significant change on free thyroxine (fT_4_) levels. The relationship between hydroxylated metabolites of PCBs (HO-PCBs) and TH status in adults was studied only in fishermen from the Baltic Sea, and no association was found (Hagmar et al. 2001a). With regard to polybrominated diphenyl ethers (PBDEs), studies investigating the thyroid-disrupting potential of these flame retardants in populations exposed to background levels are scarce, inconsistent, and limited to male populations (Bloom et al. 2008; Hagmar et al. 2001a; Julander et al. 2005; Turyk et al. 2008). On the other hand, potential effects of perfluoro-octanesulfonate (PFOS) on THs in an environmentally exposed adult population have been investigated only in one small study in which no significant association was observed with either TSH or fT_4_ (Bloom et al. 2009).

Nunavik Inuit are exposed to relatively high concentrations of PCBs and other organo-chlorines (OCs) through their seafood-based traditional diet compared with populations from southern regions of North America. Considering the thyroid-disrupting potential of these contaminants, the aim of the present study was to investigate the relationship between several PHCs and thyroid function in the Inuit adult population of Nunavik.

## Materials and Methods

### Population, study design, and data collection

We conducted a comprehensive health cross-sectional study in the Inuit population of Nunavik (Québec, Canada) from 27 August to 1 October 2004. The target population of this study was permanent Inuit residents of Nunavik ≥ 18 years of age. We used a stratified random sampling of private Inuit households, with the community being the stratification variable. The study was approved by the Comité d’éthique de la recherche de l’Université Laval and the Comité d’éthique de santé publique du Québec. All participants provided written consent before taking part in the study.

Several self-administered and interviewer-completed questionnaires were used to obtain information regarding demographics, lifestyle habits, and nutrition. In addition, individuals were asked to participate in a clinical session where blood samples were taken and physical measurements were performed. Overall, the participation rate for the different collection instruments was approximately 50%, for a total of 1,056 participants. We assessed contaminant concentrations on 889 plasma samples. Because TH concentrations fluctuate greatly during pregnancy, we excluded pregnant women from the present study (*n* = 23). Non-Inuit (*n* = 20) and individuals using medication for thyroid diseases (*n* = 19) were also excluded.

### Demographic, clinical, and nutritional variables

We obtained data related to education, income, and cigarette consumption through an interviewer-administered questionnaire; information on alcohol consumption was obtained from a questionnaire completed confidentially by the participant. We obtained data regarding medication use in the 6 months preceding the study from medical charts. Among participants with quantified concentrations of contaminants, 729 had completed questionnaires regarding sociodemographic information, and 628 had records of medication consumption. Data on fish consumption were obtained using a food frequency questionnaire from 536 participants with quantified PHC concentrations. Details regarding the calculation of fish consumption are described in Supplemental Material available online (doi:10.1289/ehp.0900633.S1 via http://dx.doi.org/).

### Laboratory procedures

Blood samples (60 mL) were collected using a venous catheter from an antecubital vein. Tubes were centrifuged within 3 hr of collection and plasma was isolated, aliquoted, and frozen at −80°C before clinical biochemistry and contaminant analyses. We analyzed plasma samples for PCBs and their hydroxylated (HO) and methylsulfonyl (MeSO_2_) metabolites, OCPs, halogenated phenolic compounds, and PBDEs according to a multiresidue gas chromatography/mass spectrometry (MS) method. Plasma samples were also analyzed for PFOS using liquid chromatography/MS and for DLCs using a reporter gene bioassay. See the Supplemental Material (doi:10.1289/ehp.0900633.S1) for details of these laboratory procedures.

We report plasma concentrations of PCBs, PBDEs OCPs, and DLCs on a lipid basis. Cholesterol and triglyceride analyses were conducted using a Hitachi 917 auto analyzer and reagents from Roche Diagnostics (Indianapolis, IN, USA). Concentrations of total plasma lipids were estimated from cholesterol and triglyceride levels according to the formula developed by Phillips et al. (1989).

We determined selenium concentrations in whole blood by inductively coupled plasma MS. Samples were diluted in ammonium hydroxide and then analyzed on an Elan 6000 instrument (Perkin Elmer-Sciex, Ontario, Canada). The limit of detection (LOD) was 0.09 μmol/L, and the coefficient of variation (CV) was 7.9% for a reference specimen (3.3 μmol/L) analyzed on 10 different days.

Analyses of serum thyroid-stimulating hormone (TSH), fT_4_, tT_3,_ and thyroxine-binding globulin (TBG; the major transport protein of TH), were performed using radioimmunoassay methods. We measured TSH, fT_4_, and tT_3_ on the Elecsys 1010/2010 analyzers and the Modular Analytics E170 immunoassay module (Roche Diagnostics). The interassay CVs were 2.0% for TSH, 4.7% for fT_4_, and 3.3% for tT_3_. TBG, determined using the Clinical Assays GammaDab system commercialized by DiaSorin (Stillwater, MN, USA), had an inter-assay CV of 3.6%.

### Statistical analysis

We determined potential effects of contaminants on thyroid status for PHCs with a detection frequency > 70%, except for BDE-47, which was detected in 57% of samples. We included BDE-47 regardless of detection frequency because of the growing concern regarding the rapid increase in concentrations measured in human samples in North America and because of its toxicologic properties. Parameters were estimated randomly from a log-normal distribution using the maximum likelihood estimation for samples with concentrations of contaminants below LODs of the analytical procedures. The multiple imputation method was an adequate statistical approach compared with the assignment of a value equal to LOD/2 when non detected values represent > 5% of the sample (Lubin et al. 2004). Effects of PCB congeners were assessed individually or as a group for mono-*ortho* PCBs (PCBs 74, 105, 118, and 156). We examined the relationship of environmental contaminants with THs (TSH, fT_4_, and tT_3_) and TBG by simple and multiple linear regression models. Contaminant concentrations and thyroid parameters were all log-transformed to satisfy criteria of normality. Five participants with extreme values of TSH were excluded.

Interaction terms between sex and all contaminants in relation to TH concentrations were tested in full models. Because all interaction terms were nonsignificant, we examined effects of PHCs on the TH homeo stasis in men and women jointly. We tested covariates known as risk factors for thyroid diseases or suspected to be associated with TH parameters to determine if they should be included in regression models. Covariates associated at a *p*-value ≤ 0.10 were included in multiple regression models to assess their confounding influence. Covariates are presented in Table 1 and are expressed as continuous variables, except where noted. Some participants declared taking medication known to alter thyroid function tests, including estrogen formulations, heparin, beta-blockers, furosemide, nonsteroidal anti-inflammatory agents, glucocorticoids, anticonvulsants, dopaminergics, and carboxamides. We created a dichotomous variable to account for the consumption of those medications. Covariates that modified the regression coefficient of the contaminants with any of the THs by > 10% were included in adjusted models. However, considering that data concerning alcohol and fish consumption were missing for 42 and 92 participants, respectively, and that their confounding effects were observed for a small proportion of associations, their inclusion in regression models was not systematic. We evaluated multicollinearity for all multiple regression models. Also, a contaminant in each family of PHCs (PCB-153, BDE-47, and PFOS) was selected and introduced together in multiple models to assess their influence on the associations with the contaminants under study.

We used sample weights to account for the complex sampling method and to obtain appropriate SEs and *p*-values. We considered a bilateral *p*-value < 0.05 statistically significant. Statistical analyses were performed with SAS (version 9.1; SAS Institute Inc., Cary, NC, USA) and SUDAAN software (version 9.0.1; Research Triangle Institute, Research Triangle Park, NC, USA).

## Results

Sociodemographic, personal, and clinical characteristics of participants are presented in Table 1. Most participants had TH concentrations in the laboratory test euthyroid range (TSH, 95.0%; fT_4_, 96.5%; tT_3_, 99.2%; TBG, 86.3%). Plasma concentrations of PHCs measured in adult Inuit are shown in Table 2. Concentrations of all PCB congeners were highly intercorrelated (*r* = 0.71–0.98; *p* < 0.001) and were strongly correlated with their metabolites [for the sum of PCBs (∑PCBs) and ∑HO-PCBs, *r* = 0.94; for ∑PCBs and ∑MeSO_2_-PCBs, *r* = 0.90; *p* < 0.001]. ∑PCB was less strongly correlated with DLC concentration (*r* = 0.69; *p* < 0.001) but still highly correlated with concentrations of OCPs (*r* = 0.85–0.95; *p* < 0.001). Pentachlorophenol was not correlated with any contaminant. Plasma concentrations of BDE-47 were moderately correlated with BDE-153 levels (*r* = 0.36; *p* < 0.001) but were not correlated with those of other contaminants. Plasma concentrations of PFOS were moderately correlated with those of PCB congeners and their metabolites (*r* = 0.47–0.55; *p* < 0.001), other OCs (*r* = 0.36–0.51; *p* < 0.001), and BDE-153 (*r*= 0.22; *p*< 0.001) but not with BDE-47.

Associations between contaminant plasma concentrations and TH concentrations are presented in Tables 3–5. Associations with further adjustment for exposure to other families of contaminants are presented in Supplemental Material, Tables S1–S3 (doi:10.1289/ehp.0900633.S1). We observed significant negative associations between the more highly chlorinated PCB congeners and TSH in multiple linear regression models (Table 3). We found no association between concentrations of PCB congeners or their groupings and fT_4_ levels in adjusted models. Fourteen PCB congeners, ∑PCBs, and the mono-*ortho* PCB group, as well as the DLCs, were negatively associated with tT_3_ concentrations in multiple models. Further adjustment for fish consumption weakened associations with some PCB congeners and groupings. We observed significant negative associations between TBG concentrations and less-chlorinated PCBs as well as the mono-*ortho* PCB group grouping in adjusted models. The significance of some associations disappeared after controlling for fish and alcohol consumption. Also, adjustment for other families of contaminants weakened some associations with TSH, tT_3_, and TBG while revealing significant negative associations of PCB-118 and mono-*ortho* PCB congeners with fT_4_ concentrations [Supplemental Material, Table S1 (doi:10.1289/ehp.0900633.S1)].

HO-PCBs and MeSO_2_-PCBs were not significantly associated with TSH and fT_4_ concentrations in multiple models, except for HO-PCB-199 and HO-PCB-208, which were negatively related to TSH (Table 4). Seven HO-PCBs, the ∑HO-PCBs, three MeSO_2_-PCBs, and the ∑MeSO_2_-PCBs were all significantly associated with a decrease in tT_3_ concentrations. We also found significant negative associations between TBG and five HO-PCBs and the ∑HO-PCBs. Only the associations for HO-PCB-107 and ∑HO-PCBs remained significant after controlling for fish consumption. Control for the exposure to other families of contaminants weakened some associations with tT_3_ and TBG but revealed significant negative associations of all MeSO_2_-PCBs metabolites and their sum (∑MeSO_2_-PCBs) with fT_4_ levels [Supplemental Material, Table S2 (doi:10.1289/ehp.0900633.S1)].

Plasma concentrations of OCPs were not significantly associated with TSH levels (Table 5). Also, among OCPs, only hexachlorobenzene (HCB) concentrations were negatively associated with fT_4_ circulating levels, whereas this compound and β-hexachlorocyclohexane (β-HCH) were negatively related to tT_3_ concentrations. TBG concentrations were negatively associated with all OCPs, except for pentachloro phenol in multiple models. Further adjustments for alcohol consumption had no impact on the relationship between OCPs and TH parameters, except for TBG, for which only the associations with HCB and β-HCH remained significant. Adjustment for other families of contaminants revealed a significant positive association between β-HCH and TSH levels [see Supplemental Material, Table S3 (doi:10.1289/ehp.0900633.S1)]. Pentachlorophenol plasma concentrations were not associated with any of the TH parameters. Among PBDEs, only BDE-47 was positively associated with tT_3_ concentrations in multiple models. This association weakened after controlling for fish consumption. We observed a significant negative association for PFOS concentrations with TSH, tT_3_, and TBG circulating levels, whereas we found a positive association with fT_4_ levels. Adjustment for other potential confounders and families of contaminants weakened the association with tT_3_ concentrations.

## Discussion

Our findings in the present study suggest modifications of the thyroid parameters in adult Inuit by PHCs. This is the first large-scale study investigating the effect of PFOS on TH homeostasis in an environmentally exposed adult population. We observed significant negative associations of PFOS concentrations with TSH, tT_3_, and TBG levels, whereas we found a positive association with fT_4_ concentrations. Furthermore, although exposure to BDE-47 in Inuit adults is lower than that in other North American populations, we found a positive association of tT_3_ with increasing concentrations BDE-47. Several PCBs, their metabolites, and OCPs are negatively associated with tT_3_.

Thyroid parameters in Inuit adults were mostly affected by exposure to PFOS. Indeed, we found a negative association of PFOS concentrations with circulating TSH, tT_3,_ and TBG levels and a positive relationship with fT_4_. Toxicologic studies dedicated to investigating the thyroid-disrupting properties of PFOS are rare and focus mainly on maternal and fetal thyroid functions. In adult rats, PFOS was shown to compete with fT_4_ for transport proteins (Chang et al. 2008), leading to a transient increase in fT_4_ and decrease in TSH concentrations. However, these authors also found a nontransient reduction in tT_4_ and tT_3_, with the latter being less affected. These results in laboratory animals are consistent with perturbations we observed on the TH homeostasis in Inuit, except that effects observed on the circulating concentrations of fT_4_ and TSH do not seem to be transient in our study.

Although exposure to PBDEs in Inuit men and women is lower than levels reported in other North American populations, we found a positive association of tT_3_ with BDE-47 but no association with BDE-153. These results differ from those observed in men from other fish-eating communities. Exposure to PBDEs was positively related to tT_4_, fT_4_, and urinary T_4_, as well as with reverse T_3_ levels, and negatively related to tT_3_ and TSH in Great Lake fish consumers (Turyk et al. 2008). In addition, fT_4_ and TSH were not associated with PBDEs in New York sport fish consumers (Bloom et al. 2008), whereas a negative association with TSH, but not fT_4,_ was reported in anglers from the Baltic Sea (Hagmar et al. 2001a). No other epidemiologic study assessed the potential effect of PBDEs on women’s thyroid function. So far, associations observed in human studies are not consistent with effects observed in animal models. Exposure to PBDE commercial mixtures was systematically associated with decreased T_4_ concentrations, subtle variations of T_3,_ and unchanged TSH concentrations in rodents (Hallgren et al. 2001; Zhou et al. 2001).

We observed similar associations between thyroid function parameters and concentrations of PCB congeners, their metabolites, and concentrations of OCPs and DLCs. This is not surprising because the Inuit population is exposed to a complex mixture of OCs, with concentrations of compounds being highly inter correlated. Consequently, it is almost impossible to isolate specific effects of PCB congeners and their metabolites, as well as OCPs, on the thyroid system in the context of epidemiologic studies in this population. TSH concentrations were lowered only by exposure to the most chlorinated PCB congeners and HO metabolites. Total T_3_ concentrations were negatively associated with almost all PCB congeners and their metabolites, as well as with OCPs and DLCs. Circulating levels of TBG, the predominant TH carrier protein in humans, were negatively associated with the less chlorinated PCBs, all HO-PCBs, and almost all other OCPs. Adjustment for other families of contaminants weakened some associations of PCB congeners and their metabolites as well as OCPs with TH parameters, probably because introduction of contaminants in regression models that were not associated with TH parameters added random variation in statistical models.

Most epidemiologic studies examining the relations between PCBs and thyroid function in adults reported no association with TSH and fT_4_ (Hagmar et al. 2001a, 2001b; Meeker et al. 2006; Rylander et al. 2006; Sala et al. 2001). Associations with tT_4_ and/or tT_3_ were reported to be negative in half of the studies, whereas the remaining studies found no relation with these hormones (Abdelouahab et al. 2008). Interestingly, associations of PCBs with total T_3_ and T_4_ were never positive. Therefore, results from the present study are in accordance with most previous findings in human adults. The independent effect of PCB exposure on tT_3_ circulating levels was previously reported in American men from the general population of Boston, Massachusetts (Meeker et al. 2006), in obese men that underwent a weight-loss program (Pelletier et al. 2002), and in fishermen’s wives who consumed PCB-contaminated fish from the Baltic Sea (Hagmar et al. 2001b). Although the thyroid function of pregnant women and children may not be comparable because they may be more vulnerable to thyroid dysfunction, isolated effects on T_3_ concentrations have also been observed in pregnant Canadian women (Takser et al. 2005), as well as in children from Germany and Spain (Alvarez-Pedrerol et al. 2008; Osius et al. 1999).

Toxicologic studies with animal models have demonstrated differential mechanisms by which PCBs and OCPs induce perturbations of the thyroid system, mainly by reducing fT_4_ concentrations (Brouwer et al. 1995; Cheek et al. 1999; van Raaij et al. 1993). DLCs, including some PCB congeners, bind to the aryl hydrocarbon receptor (AhR) and activate the hepatic uridine diphosphate glucuronyltransferase (UDP-GT) enzymes responsible for T_4_ glucuronidation, resulting in T_4_ excretion in bile. Non-DL-PCBs do not cause AhR-dependent effects but induce microsomal cytochrome P450 enzymes 2B and 3A that may also reduce circulating T_4_ levels (Khan et al. 2002). However, these mechanisms do not explain the selective effect of most PCBs and OCPs on tT_3_ concentrations observed in this study and others. One possible explanation could be that some of the compounds may affect peripheral deiodination of T_4_ by inactivation of deiodinase enzymes (Schuur et al. 1998), leading to reduced T_3_ levels. Also, some PCBs or their metabolites may alter the activities (activation or inactivation) of specific T_3_- or T_4_-directed hepatic UDP-GT enzymes, affecting principally the circulating levels of T_3_ (Vansell and Klaassen 2002). However, we are unable to adequately evaluate those hypotheses because we did not measure tT4 in the course of this study. Another potential mechanism could be that those compounds primarily affect TBG concentrations, leading to a reduction in the TH-binding capacity of serum. Because serum THs are mainly bound to carrier proteins and TBG is the main transporter (75% T_4_ and 70% T_3_) (Schussler 2000), such alterations could reduce total TH concentrations as we observed with tT_3_ in this study. Interestingly, individuals with inherited or acquired TBG excess have elevated total T_3_ and T_4_ concentrations, whereas those with TBG deficiency have decreased concentrations (Bartalena and Robbins 1992). Nevertheless, those individuals are still considered euthyroid because of their normal TSH and fT_4_ levels. Similarly, PCBs or OCPs may affect TBG levels and consequently tT_3_ and tT_4_ without modifying TSH and fT_4_ concentrations. This potential mechanism could explain why most epidemiologic studies among adults reported negative associations with circulating tT_4_ and/or tT_3_ but no association with TSH and fT_4_.

We could not compare our results on the effect of PCBs on TBG concentrations in adults because TBG quantification was not performed in other epidemiologic studies. Explanations given for the negative associations between several PHCs and TBG concentrations observed in two previous studies (Dallaire et al. 2008, 2009) and this one are unclear, but they suggest a decrease of TBG synthesis by the liver or an increase in catabolism. Unfortunately, such mechanisms cannot be corroborated by toxicologic data because studies have focused on the competitive binding assessment of contaminants with T_4_, without paying attention to their possible ability to modify transport protein synthesis.

In the present study, exposure to HCB was associated with reduced fT_4_ concentrations. Exposure to HCB was also related to a concomitant decline in tT_3_ and TBG, without association with TSH concentrations. HCB-treated rats usually showed a decline in fT_4_ and tT_4_ levels with no change in tT_3_ and inconsistent effects on TSH concentrations (Alvarez et al. 2005; van Raaij et al. 1993). Activation of hepatic UDP-GT enzymes by HCB, followed by T_4_ glucuronidation and subsequent biliary excretion, is the main mechanism explaining hypothyroidism state in rats. In humans, considering our findings, this contaminant may induce a reduction in TBG concentrations, which decreases the T_3_ and potentially T_4_ binding capacity of serum, whereas reduction of fT_4_ may be related to UDP-GT enzymes induction in the liver.

HO-PCBs and pentachlorophenol were not associated with a significant reduction in fT_4_ concentrations. Only one study has investigated the effect of HO-PCBs on thyroid homeostasis of fishermen consuming fish from the Baltic Sea, and no association was found (Hagmar et al. 2001a). Those compounds have been shown to be potent competitive inhibitors of T_4_ binding to transthyretin (van den Berg 1990), one of the TH carrier proteins, leading to increased T_4_ metabolism. However, because transthyretin contributes to only one-fifth of bound T_4_, this mechanism is less likely to have an impact on fT_4_ concentrations in adults. On the other hand, such a mechanism could be important in fetuses and neonates because transthyretin is involved in T_4_ placental transfer (McKinnon et al. 2005) and transport through the blood–brain barrier (Schreiber et al. 1995).

Results from the present study demonstrate that PHCs differentially alter the circulating levels of THs in humans. It is therefore extremely difficult to predict the total effect of exposure to a complex mixture of thyroid- disrupting chemicals on thyroid status. Assessments of effects of exposure to mixtures on the thyroid systems in animal models are scarce. In male rats, additivity under estimated the effect of a mixture of 16 OCs, lead, and cadmium on different thyroid end points (Wade et al. 2002). Relationship between tT4 and exposure to a mixture of dioxins, furans, and PCBs in rats was shown to be additive at the lowest doses, but synergism was observed at higher doses (Crofton et al. 2005). From our results and those of others, there is increasing evidence that emerging persistent chemicals such as PBDEs and PFOS are also thyroid disrupting and should be included in future studies aiming to assess the potential effects of persistent organic pollutants on the thyroid system.

This study has several strengths that are worth mentioning. First, we used a sample-based sampling design and recruited a large number of participants. We considered confounding effects for an important number of covariates, including medication use and concomitant exposure to other PHCs. However, we were unable to evaluate the individual effect of OCs on TH status by controlling for other OCs because of the high intercorrelations between these compounds. One limitation of this study is that we did not use the equilibrium dialysis method that is less binding- protein dependent for fT4 quantification.

In summary, we observed a significant reduction of tT_3_ with exposure to several PCBs and their metabolites, as well as exposure to some OCPs. A large number of PHCs seem to modify circulating levels of TBG, the main TH transport protein in humans. In addition, we found that exposure to BDE-47 and PFOS, at levels observed in several populations worldwide, appear to modify levels of TH parameters. However, other epidemiologic, animal, and *in vitro* studies are needed to corroborate these results. Because most Inuit participants had a thyroid status within the euthyroid range, it is not clear if the observed effects could be associated with increasing disease burden in adults.

## Figures and Tables

**Table 1:** Characteristics of adult Inuit of Nunavik.

Characteristic	No.	Percentage	Mean ± SD	Range
Sex (%)				
Male	245	39.3		
Female	378	60.7		
Menopause (% among females)				
Yes	52	12.5		
No	363	87.5		
Age (years)	623		36.8 ± 13.9	18–73
Body mass index (kg/m^2^)	623		27.4 ± 5.7	17.2–48
Selenium (μmol/L)	622		4.4 ± 3.0	1.5–30.0
No. of cigarettes/day (%)				
0	136	21.8		
1–10	187	30.0		
11–24	226	36.3		
≥ 25	74	11.9		
Frequency of alcohol consumption (%)				
Never or yearly	287	49.4		
Monthly	148	25.8		
Weekly	101	17.4		
Daily	45	7.8		
Fish consumption (g/day)^a^	563		54.7 ± 75.1	0–665.6
Marine mammal consumption (g/day)^a^	561		27.1 ± 44.4	0–398.7
Education (%)				
None	46	7.4		
Elementary school	99	15.9		
High school	394	63.2		
University	84	13.5		
TSH (mIU/L)^b^	623		1.18 ± 0.79	0.06–6.45
fT_4_ (pmol/L)^b^	621		15.5 ± 2.1	9.3–22.9
tT_3_ (nmol/L)^b^	621		2.14 ± 0.33	1.21–3.39
TBG (μg/mL)^b^	622		24.0 ± 6.0	8.0–62.8
Medication use in the last 6 months (%)				
Yes	106	17.0		
No	517	83.0		

a Expressed on an annual basis.

b Laboratory euthyroid reference range: TSH, 0.27–4.20 mIU/L; fT_4_, 12–22 pmol/L; tT_3_, 1.3–3.1 nmol/L ; TBG, 13–30 μg/mL.

**Table 2:** Plasma concentrations of PHCs in Inuit participants.

Analyte	No.	Percent detected	Geometric mean	95% CI	Range
PCB congener^a^					
PCB-74	623	95.0	8.92	8.15–9.75	< LOD–218.77
PCB-99	623	99.7	25.53	23.38–27.87	< LOD–849.53
PCB-105	623	78.4	3.49	3.10–3.93	< LOD–100.74
PCB-118	623	99.8	22.76	20.79–24.91	< LOD–497.09
PCB-138	623	100	78.03	71.41–85.26	3.66–1388.42
PCB-146	623	98.7	22.15	20.14–24.36	< LOD–589.00
PCB-153	623	100	180.06	163.71–198.06	5.69–5805.15
PCB-156	623	87.7	6.70	5.97–7.51	< LOD–330.97
PCB-163	623	99.2	26.88	24.39–29.63	< LOD–807.77
PCB-170	623	99.4	25.88	23.42–28.61	< LOD–869.17
PCB-172	621	78.7	3.95	3.49–4.47	< LOD–180.20
PCB-177	623	82.7	3.96	3.56–4.41	< LOD–89.13
PCB-178	623	90.1	7.83	7.01–8.73	< LOD–235.60
PCB-180	623	100	93.28	84.53–102.93	3.25–3510.34
PCB-183	623	93.5	9.16	8.35–10.06	< LOD–177.74
PCB-187	623	99.8	37.98	34.67–41.61	1.18–790.66
PCB-194	623	95.1	15.74	13.94–17.77	< LOD–945.49
PCB-201	623	96.1	15.88	14.19–17.78	< LOD–772.27
PCB-203	623	89.4	7.93	7.08–8.88	< LOD–294.20
PCB-206	623	78.2	4.02	3.51–4.59	< LOD–275.81

PCB grouping^a^					
∑PCBs	621		635.98	579.33–698.18	31.47–14260.92
Mono-*ortho* PCB	623		45.24	41.34–49.51	1.80–866.67
DLCs^b^	607	72.6	10.62	10.04–11.23	< LOD–139.30

HO-PCB^c^					
3-HO-PCB-138	562	89.0	30.38	27.17–33.98	< LOD–980
3-HO-PCB-153	536	97.5	36.27	32.38–40.63	< LOD–1,500
4-HO-PCB-107	566	99.5	173.21	158.67–189.08	< LOD–3,200
4-HO-PCB-146	536	99.5	136.88	123.26–152.01	< LOD–4,900
4-HO-PCB-163	567	94.0	11.20	10.20–12.29	< LOD–590
4-HO-PCB-172	550	96.6	19.19	17.30–21.27	< LOD–790
4-HO-PCB-187	567	99.8	157.69	143.67–173.07	< LOD–4,300
4-HO-PCB-199	568	94.5	19.10	17.20–21.22	< LOD–1,200
4-HO-PCB-202	566	87.9	8.42	7.66–9.26	< LOD–340
4-HO-PCB-208	568	75.0	5.64	5.13–6.21	< LOD–340
∑HO-PCBs	536		642.02	584.52–705.18	39.44–14,610

MeSO_2_-PCB^c^					
3-MeSO_2_-PCB-49	573	93.4	26.39	23.77–29.30	< LOD–910
3-MeSO_2_-PCB-87	573	76.5	11.23	10.04–12.57	< LOD–610
3-MeSO_2_-PCB-101	575	86.9	18.50	16.55–20.68	< LOD–920
∑MeSO_2_-PCBs	573		57.54	51.71–64.03	< LOD–2,270

OC^a^					
*p,p*′-DDE	623	100	477.78	441.70–516.81	12.85–8306.48
HCB	623	99.7	61.36	56.38–66.78	< LOD–1245.97
Pentachlorophenol^c^	567	100	801.43	758.25–847.07	140–18,000
β-HCH	622	95.9	8.33	7.70–9.02	< LOD–201.94

PBDE^a^					
BDE-47	623	57.3	2.16	1.84–2.54	< LOD–343.45
BDE-153	623	73.8	2.05	1.85–2.27	< LOD–75.74

Perfluorinated compound					
PFOS^c^	621	100	18,280	17,190–19,440	480–470,000

Abbreviations: β-HCH, β-hexachlorocyclohexane; DDE, *p,p*′-dichlorodiphenyldichloroethylene; HCB, hexa chlorobenzene.

a Lipid standardized concentrations (μg/kg).

b Concentrations in picograms toxic equivalents per gram lipids.

c Concentrations on a wet weight basis (ng/L).

**Table 3:** Linear regression models of THs and TBG plasma levels with PCB and DLC concentrations (wet weight basis).

		TSH		fT_4_		T_3_		TBG	
Analyte	No.	Unadjusted β	Adjusted β	Unadjusted β	Adjusted β	Unadjusted β	Adjusted β	Unadjusted β	Adjusted β
PCB congener									
PCB-74	623	0.052**	0.014	−0.019#	−0.004	−0.028#	−0.020*	−0.001	−0.036#
PCB-99	623	0.054**	0.022	−0.015**	0.003	−0.025#	−0.012	−0.004	−0.034**
PCB-105	623	0.039**	0.017	−0.015#	−0.004	−0.021#	−0.018**	−0.001	−0.030#
PCB-118	623	0.058**	0.027	−0.021#	−0.009	−0.030#	−0.028**	−0.005	−0.051#
PCB-138	623	0.046*	0.000	−0.016#	0.000	−0.027#	−0.015	−0.001	−0.028*
PCB-146	623	0.035*	−0.023	−0.016#	−0.004	−0.028#	−0.020**	−0.000	−0.026*^a^
PCB-153	623	0.033	−0.024	−0.014**	0.001	−0.027#	−0.017*^a^	0.001	−0.021
PCB-156	623	0.021	−0.035	−0.012**	−0.003	−0.022#	−0.014*^a^	0.005	−0.003
PCB-163	623	0.039*	−0.009	−0.016#	−0.007	−0.028#	−0.023**	0.000	−0.024*^a^
PCB-170	623	0.026	−0.040	−0.014#	−0.004	−0.026#	−0.016*^a^	0.007	−0.000
PCB-172	621	0.025	−0.019	−0.010**	0.000	−0.022#	−0.014*	0.004	−0.005
PCB-177	623	0.036*	−0.006	−0.014**	−0.002	−0.024#	−0.015*	0.001	−0.018*^b^
PCB-178	623	0.021	−0.039	−0.014**	−0.007	−0.025#	−0.019**	0.003	−0.008
PCB-180	623	0.020	−0.059*	−0.012**	0.001	−0.025#	−0.011	0.007	−0.000
PCB-183	623	0.031	−0.024	−0.012**	0.006	−0.025#	−0.011	0.003	−0.017
PCB-187	623	0.040*	−0.013	−0.017#	−0.006	−0.029#	−0.018*^a^	0.001	−0.023
PCB-194	623	0.008	−0.069**	−0.011**	−0.003	−0.021#	−0.012*^c^	0.007	0.005
PCB-201	623	0.016	−0.051*	−0.012**	−0.003	−0.024#	−0.016*^a^	0.006	−0.003
PCB-203	623	0.008	−0.069**	−0.010**	0.003	−0.021#	−0.008	0.006	−0.000
PCB-206	623	0.001	−0.075**	−0.009**	0.004	−0.017#	−0.003	0.011	0.013

PCB grouping									
∑PCBs	623	0.029	−0.040	−0.015#	−0.003	−0.029#	−0.020*	0.003	−0.017
Mono-*ortho* PCBs	623	0.051**	0.009	−0.021#	−0.012	−0.031#	−0.028#	0.001	−0.037**
DLCs	607	0.092#	0.019	−0.017*	0.007	−0.043#	−0.027*	−0.002	−0.027

All models adjusted for sex, age, body mass index, plasma lipids, cigarette consumption (cigarettes/day), and education. Associations remained significant after adjusting for fish and alcohol consumption, unless noted.

a Borderline significant after adjusting for fish consumption (0.05 < *p* < 0.07).

b Not significant after adjusting for alcohol consumption.

c Not significant after adjusting for fish consumption.

* *p* ≤ 0.05.

** *p* < 0.01.

# *p* < 0.001.

**Table 4:** Linear regression models of THs and TBG plasma levels with HO-PCB and MeSO_2_-PCB concentrations (wet weight basis).

		TSH		fT_4_		T_3_		TBG	
Analyte	No.	Unadjusted β	Adjusted β	Unadjusted β	Adjusted β	Unadjusted β	Adjusted β	Unadjusted β	Adjusted β
HO compound									
3-HO-PCB-138	562	0.033*	−0.009	−0.014**	−0.003	−0.031#	−0.027#	−0.002	−0.021*^a^
3-HO-PCB-153	536	0.029	−0.017	−0.009	0.008	−0.028#	−0.023**	−0.001	−0.027*^b^
4-HO-PCB-107	566	0.020	−0.020	−0.011*	0.009	−0.029#	−0.013	−0.016	−0.052#
4-HO-PCB-146	536	0.025	−0.025	−0.014**	−0.003	−0.028#	−0.018*^b^	0.000	−0.023*^b^
4-HO-PCB-163	567	0.023	−0.052	−0.015**	−0.002	−0.032#	−0.020*	−0.004	−0.023
4-HO-PCB-172	550	0.027	−0.028	−0.012*	0.002	−0.031#	−0.024**	−0.001	−0.021
4-HO-PCB-187	567	0.023	−0.052	−0.014*	−0.000	−0.033#	−0.022*	−0.006	−0.029*^b^
4-HO-PCB-199	568	0.008	−0.084**	−0.010*	0.003	−0.027#	−0.014	0.001	−0.008
4-HO-PCB-202	566	0.019	−0.056	−0.013*	−0.001	−0.033#	−0.023**	−0.001	−0.016
4-HO-PCB-208	568	0.006	−0.084*	−0.011*	0.002	−0.027#	−0.011	0.003	−0.007
∑HO-PCBs	536	0.035	−0.025	−0.015*	0.000	−0.033#	−0.025*	−0.002	−0.034**

MeSO_2_-PCB									
3-MeSO_2_-PCB-49	573	0.040*	−0.007	−0.018#	−0.008	−0.028#	−0.019**	0.003	−0.011
3-MeSO_2_-PCB-87	573	0.040*	0.002	−0.018#	−0.010	−0.026#	−0.017**	0.003	−0.011
3-MeSO_2_-PCB-101	575	0.037*	−0.009	−0.017#	−0.008	−0.026#	−0.016*	0.006	−0.004
∑MeSO_2_-PCBs	573	0.041*	−0.006	−0.018#	−0.009	−0.028#	−0.018**	0.005	−0.008

All models adjusted for sex, age, body mass index, plasma lipids, cigarette consumption (cigarettes/day), and education. Associations remained significant after adjusting for fish and alcohol consumption unless noted.

a Not significant after adjusting for fish consumption.

b Borderline significant after adjusting for fish consumption (0.05 < *p* < 0.07).

* *p* ≤ 0.05.

** *p* < 0.01.

# *p* < 0.001.

**Table 5:** Linear regression models of THs and TBG plasma levels with OCPs, PBDE, and PFOS concentrations (wet weight basis).

		TSH		fT_4_		T_3_		TBG	
Analyte	No.	Unadjusted β	Adjusted β	Unadjusted β	Adjusted β	Unadjusted β	Adjusted β	Unadjusted β	Adjusted β
OC									
*p,p*′-DDE	623	0.051*	0.012	−0.021#	−0.007	−0.030#	−0.016	−0.001	−0.030*^a^
HCB	623	0.048*	0.010	−0.026#	−0.017**	−0.033#	−0.030#	−0.006	−0.054#
Pentachlorophenol	567	0.008	−0.001	0.022**	0.014	−0.008	−0.009	−0.037*	−0.021
β-HCH	622	0.081#	0.047	−0.022#	−0.008	−0.032#	−0.028**	−0.007	−0.051#

PBDE									
BDE-47	623	0.000	−0.002	0.002	0.001	0.010#	0.008**^b^	0.002	0.003
BDE-153	623	0.017	−0.009	0.007	0.009	−0.001	0.005	−0.002	0.005

Perfluorinated compound									
PFOS	621	−0.017	−0.102*	0.004	0.014*	−0.030#	−0.017*	−0.026*	−0.034**

All models adjusted for sex, age, body mass index, plasma lipids, cigarette consumption (cigarettes/day), and education. Associations remain significant after adjusting for fish and alcohol consumption unless noted.

a Borderline significant after adjusting for alcohol consumption (0.05 < *p* < 0.07).

b Borderline significant after adjusting for fish consumption (0.05 < *p* < 0.07).

* *p* ≤ 0.05.

** *p* < 0.01.

# *p*< 0.001.
